# Essentials of cardiac MRI in clinical practice

**DOI:** 10.1186/1532-429X-18-S1-T10

**Published:** 2016-01-27

**Authors:** Albert D Piersson

**Affiliations:** Radiology, Tamale Teaching Hospital, Accra, Ghana

## Background

Cardiac MRI (CMRI) is considered the "gold" standard for non-invasive characterization of cardiac function and viability as it allows the assessment of regional and global cardiac function, mass, volumes, myocardial perfusion, and tissue characterization. With its excellent soft-tissue differentiation, CMRI is the most important imaging modality for differentiating tumour from thrombus, distinguishing malignant from benign masses, and helps determine the extent of pericardial and myocardial invasion in cardiac masses. It is also indicated for evaluating cardiomyopathies, myocardial ischaemia or infarction, coronary artery disease, valvular disease, and complex congenital abnormalities.

## Methods

A literature review was conducted to evaluate the MRI pulse sequences that are applicable in CMRI, common artefacts, and breathing techniques.

## Results

The use of turbo spin-echo (TSE) is ideal for black blood imaging as it is used for morphological cardiac imaging, and the hydrogen spins exposed to both the refocusing pulse and excitation signal at the same location produce the signal void which is advantageous for differentiating the "black" blood and soft-tissues i.e. fat (white) or thrombus (gray). However, even though TSE sequences can permit acquisition of single slices during a breath hold, the use of single-shot (SS)-TSE sequences may be recommended as they are even faster permitting the acquisition of a complete stack of image slices during one breath-hold. The trade-off is that SS-TSE provides less contrast than TSE sequences. Bright blood imaging is usually acquired using steady-state-free-precession (SSFP) as it yields a higher signal-to-noise ratio (SNR) of the "white" blood as opposed to the TSE sequences. Other pulse sequences that can be employed to complement these sequences include phase contrast velocity-encoded (PC-VE) sequences and 3D contrast-enhanced magnetic resonance angiogram (CE-MRA) which allow for volume and velocity measurements. Artefacts in CMRI are often due to superimposition of respiratory and cardiac motion during the imaging time. Whereas ECG gating can be used to compensate for cardiac motion as it is more precise and usually produces a superior result, on the other hand, breath-hold techniques significantly reduce respiratory artefacts. However, in patients who cannot perform breath-hold or in severe arrhythmia patients who might require free breathing method, techniques i.e. time-of-flight (TOF)-magnetic resonance angiogram (MRA), phase-contrast MRA, and 3D whole-heart-SSFP can be employed.

## Conclusions

MRI has an important role to play in imaging cardiac morphology and function. Diagnostic assessment of the heart requires high quality images with excellent SNR and spatial resolution. The use of TSE for cardiac morphology and complementing with SSFP sequences are important to reveal subtle cardiac abnormalities; however by including other sequences i.e. PC-VE and 3D-MRA, detailed diagnostic information can be better revealed.

